# 4132 Do Research Studies at Oregon Health & Science University Comply with the New NIH Inclusion Across the Lifespan Policy - A “Look Back” over the last 2 Years

**DOI:** 10.1017/cts.2020.265

**Published:** 2020-07-29

**Authors:** Meredith Zauflik, Elizabeth Wenzel, Adrienne Zell, Elizabeth Eckstrom

**Affiliations:** 1 Oregon Health & Science University

## Abstract

OBJECTIVES/GOALS: This project aims to ensure that the age ranges of participants in OHSU studies for specific diseases match the demographics of the populations the diseases occur in, as mandated by the new NIH Inclusion of Individuals Across the Lifespan as Participants in Research Involving Human Subjects policy. METHODS/STUDY POPULATION: This study involves retrospective and prospective data. The retrospective phase (“Look Back”), reviewed all investigator-initiated OHSU studies between 2017 and 2018 with prospective consent that were disease related (N = 63). Age range per IRB protocol and per subject enrollment were graphically compared to disease demographics to determine if study age ranges were a “match” or “mismatch” to disease demographics (0 = mismatch, 1 = partial match, 2 = full match). This data will inform the upcoming prospective phase of the study, when the study team will reach out to primary investigators of enrolling studies with education and resources, and track whether this reduces demographic “mismatch.” RESULTS/ANTICIPATED RESULTS: Of the studies, 51 were evaluated in the “Look Back” analysis. 40 studies were full matches for age inclusion matching disease demographics (78%), 40 for disease prevalence range (78%), and 38 for enrolling subjects within the disease demographic range (74%). Studies received the lowest scores in enrolling subjects that match disease prevalence, with 19 earning full points (37%) and 17 earning 0 points (33%). Limitations include difficulty in finding and applying disease demographic and prevalence ranges. In addition, in this data, 12 of the original 63 total studies could not be scored because no subjects had been enrolled or prevalence ranges were not in line with clinical expertise. DISCUSSION/SIGNIFICANCE OF IMPACT: This study highlights that many trials exclude older subjects at the upper age ranges. Future analysis of the prospective phase of the study will allow us to assist research teams in closing these gaps and will determine the Policy’s impact on the recruitment of older adults into research.

